# Genetic epidemiology of *C9orf72* repeat expansion associated amyotrophic lateral sclerosis in Hungary

**DOI:** 10.1186/s10020-026-01465-w

**Published:** 2026-04-15

**Authors:** Zsófia Flóra Nagy, Adrienn Géresi, Zoltán Grosz, Barbara Trombitás, Margit Pál, Dominika Nagy, András Salamon, Péter Balicza, Lívia Dézsi, Péter Klivényi, Márta Széll, Mária Judit Molnár

**Affiliations:** 1 Institute of Genomic Medicine and Rare Disorders, Semmelweis University, Budapest, Hungary; 2 HUN-REN Multiomics Neurodegeneration Research Group, Budapest, Hungary; 3 Department of Medical Genetics, University of Szeged, Szeged, Hungary; 4 HUN-REN Functional Clinical Genetics Research Group, Szeged, Hungary; 5 Department of Neurology, Albert Szent-Györgyi Clinical Center, University of Szeged, Szeged, Hungary; 6 HUN-REN-SZTE Neuroscience Research Group, Hungarian Research Network, University of Szeged, Danube Neuroscience Research Laboratory, Szeged, 6725 Hungary

**Keywords:** ALS, Amyotrophic lateral sclerosis, C9orf72

## Abstract

**Background:**

Amyotrophic lateral sclerosis (ALS) is a fatal neurodegenerative disorder characterized by progressive motor neuron loss. The most common genetic cause of ALS is the hexanucleotide repeat expansion in the *C9orf72* gene, which is associated with earlier disease onset, faster progression, and an increased frequency of cognitive and psychiatric involvement. Data on population-specific characteristics of *C9orf72*-associated ALS remains limited in Central and Eastern Europe.

**Methods:**

Between 2011 and 2024, a total of 959 ALS patients fulfilling established diagnostic criteria were screened for *C9orf72* repeat expansions at two Hungarian centers. Hexanucleotide repeat expansions were analyzed using repeat-primed long-read PCR. Repeat numbers exceeding 30 were considered pathogenic. Clinical, demographic, and disease course data were retrospectively collected and analyzed.

**Results:**

Pathogenic *C9orf72* repeat expansions were identified in 63 of 959 patients, corresponding to a prevalence of 6.57% among Hungarian ALS patients. Bulbar onset was the most common presentation and was associated with faster progression and shorter survival (mean survival: 27.8 months). Cognitive impairment and psychiatric comorbidities were present in a substantial proportion of patients and were associated with slower functional decline. Regional differences in survival were observed, likely reflecting disparities in healthcare access rather than biological factors.

**Conclusions:**

This study provides the first comprehensive national characterization of *C9orf72* repeat expansion–associated ALS in Hungary, based on a genetically defined cohort assembled over 13 years. Despite limitations related to retrospective data collection and cohort size, this ethnically homogeneous dataset offers valuable insight into population-specific clinical and epidemiological features and complements larger international studies. Systematic characterization and longitudinal follow-up of genetically defined, trial-ready ALS cohorts will be essential as targeted therapies for *C9orf72*-associated ALS approach clinical implementation.

## Introduction

Amyotrophic lateral sclerosis (ALS) is a fatal neurodegenerative disease leading to the degeneration of motor neurons in the cortex, the brainstem and in the spinal cord as well. The hallmark symptoms of ALS include gradually progressive paresis of the limbs and bulbar musculature, leading to motor deficits, disability, dysphagia, and dysarthria. Furthermore, cognitive impairment develops in approximately half of patients as the disease progresses. As for the disease onset we differentiate bulbar onset cases, lower motor neuron (LMN) onset cases and upper motor neuron (UMN) onset cases (Es et al. 2017).

Prevalence of ALS has been estimated to be at ~ 6.2 per 100,000 persons, based on aggregated data from multiple countries according to a recent meta-analysis (Brown et al. 2021). Point prevalence estimates from individual studies illustrate geographic differences in diagnosis and/or varying reporting strategies, with the lower end of the spectrum being ~ 3.4 per 100,000 (e.g., Malta), while higher point prevalence estimates range around ~ 10.8 per 100,000 (e.g., certain regions of Italy) (Farrugia Wismayer et al. 2023; Pateri et al. 2023). Calculating from pooled studies this gives us around 30′000 people currently living with ALS in the European Union and in the UK together.

ALS is a genetically heterogeneous disorder encompassing both monogenic forms, such as *SOD1*-associated ALS, and oligogenic forms in which disease risk is influenced by a small number of high-effect variants or the combined effect of multiple lower-impact variants (Van et al. 2012). However, the breakthrough in ALS genetics came in 2011 when two unrelated research groups described the intronic G_4_C_2_ hexanucleotide repeat expansion in the *C9orf72* gene as the main genetic alteration behind the disease (DeJesus-Hernandez et al. 2012; Renton et al. 2012). In populations of European origin, the *C9orf72* hexanucleotide repeat expansion may explain around 40% of familial cases and around 7% of sporadic ALS cases (Renton et al. 2014).

The function of the C9orf72 protein is not fully understood yet, it is an established guanine nucleotide exchange factor, and plays a role in the regulation of autophagy (Sellier et al. 2016; Yang et al. 2016). Several mutually non-exclusive mechanisms have been proposed regarding the pathophysiological role of the repeat expansion in ALS. First, it has been hypothesized that haploinsufficiency of the gene might hinder its physiological function (DeJesus-Hernandez et al. 2012). Second, the toxic dipeptide repeats formed by the hexanucleotide sequence might overburden the neurons and might contribute to their degeneration (Gendron et al. 2013). Lastly, it has been suggested that the C9orf72 RNA molecules could cluster in RNA foci and trap other RNAs and their binding proteins thus disturbing the physiological RNA metabolism of the cell (DeJesus-Hernandez et al. 2012).

*C9orf72*-associated ALS exhibits age-dependent incomplete penetrance and marked phenotypic variability. Bulbar onset of symptoms, earlier age at onset, faster disease progression and the presence of cognitive decline (especially the occurrence of frontotemporal dementia, FTD) seems to be more common among patients harboring the *C9orf72* hexanucleotide repeat expansion (Santos Silva et al. 2024; Chiò et al. 2012).

In the era of genomic medicine precision therapy development for ALS has commenced. The first targeted therapy for genetically determined ALS was brought to the market in 2023 in the USA and in 2024 in Europe after a successful clinical trial (Miller et al. 2022). However, since *SOD1* variants only account for 1–2% of sporadic ALS cases and 20% of familial cases, not many patients are eligible for this treatment. Thus, the development of targeted *C9orf72* drugs have been launched. Several therapeutic approaches are currently in in vitro and in in vivo stages of development, such as a CRISPR-Cas based gene editing model, or a dipeptide repeat reducing agent working through the depletion of a serine-arginine-rich splicing factor (Kempthorne et al. 2025; Hautbergue et al. 2017). Some drugs have already reached the stage of clinical trials, such as a LINE-1 retrotransposon inhibitor or PIKFYVE kinase inhibitor stimulating the clearance of aggregated proteins via exocytosis (Blitterswijk et al. 2025). Hopes are high that soon a revolutionary treatment for *C9orf72* positive ALS patients will be available.

This is why we believe that mapping the population specific genetic background and epidemiology aspects of ALS is needed for the preparation of the (hopefully near) future personalized treatment of this devastating disease.

## Patients and methods

### Patients and clinical assessment

The *C9orf72* hexanucleotide repeat expansion testing was performed at two genetic laboratories: the Institute of Genomic Medicine and Rare Disorders at Semmelweis University, Budapest, and the Department of Medical Genetics at the University of Szeged. At Semmelweis University, testing has been available since 2012, and by the end of 2024 a total of 719 patients had undergone analysis. At the University of Szeged, the first assay was performed in 2013, and since then more than 240 patients have been tested. Overall, approximately 959 Hungarian patients have been examined nationwide since the identification of the *C9orf72* repeat expansion. Altogether 25% of the *C9orf72* testing was carried out at the Department of Medical Genetics at the University of Szeged, whilst 75% was performed at the Institute of Genomic Medicine and Rare Disorders at Semmelweis University.

All patients diagnosed with amyotrophic lateral sclerosis fulfilled the diagnostic criteria applicable at the time of diagnosis, including the El Escorial, Awaji-shima, and Gold Coast criteria (Carvalho and Swash August 2008; Ludolph et al. 2015; Turner 2022). Each patient underwent a comprehensive neurological examination performed by senior neurologists. A detailed family history was obtained at the time of diagnosis.

Clinical data were retrospectively collected from electronic medical records. Disease severity and functional status were assessed using the revised ALS Functional Rating Scale (ALS-FRS-R), and disease stage was determined according to the Milano–Torino Staging (MITOS) system at the time of diagnosis and at each follow-up visit.

Psychiatric comorbidities were diagnosed by expert clinicians based on relevant psychiatric examinations and assessments. Cognitive decline was assessed by Addenbrooke's Cognitive Examination and Mini Mental State Exams performed by experienced psychologists.

All participants provided written informed consent prior to inclusion in the study. The study was conducted in accordance with the principles of the Declaration of Helsinki and its subsequent amendments and was approved by the Regional Research Ethics Committee of Semmelweis University (approval number: 308/2025).

### Genetic analysis

Peripheral blood samples were collected in EDTA-containing tubes, and genomic DNA was extracted using commercially available DNA isolation kits according to the manufacturers’ protocols. Both laboratories employed a long-read repeat-primed polymerase chain reaction (RP-PCR) assay to determine the length of the G4C2 hexanucleotide repeat in the first intron of the *C9orf72* gene. Repeat expansions exceeding 30 G4C2 units were considered pathogenic.

### Statistical analysis

Descriptive and comparative statistical analyses were performed using GraphPad Prism version 10 (GraphPad Software, USA). Statistical significance was defined as a two-tailed *p* value < 0.05.

## Results

In the present study, we performed detailed clinical characterization and phenotypic analysis of 63 Hungarian ALS patients carrying a pathogenic *C9orf72* repeat expansion. Among the 959 patients tested nationwide, 63 were identified as *C9orf72* expansion carriers, corresponding to a mutation frequency of 6.57% (63/959).

All included patients fulfilled the revised El Escorial and Gold Coast diagnostic criteria for ALS (Ludolph et al. 2015; Turner 2022). In all cases, the *C9orf72* expansion was present as a fully expanded allele (> 45 G4C2 repeats).

Eighteen patients were referred from the Department of Medical Genetics of the University of Szeged, while forty-five patients were identified at the Institute of Genomic Medicine and Rare Disorders, Semmelweis University. The cohort comprised 40 females and 23 males, yielding a female-to-male ratio of 1.74:1. The mean age at disease onset was 57.7 ± 8.4 years. Further demographic data concerning our patients can be seen in Table 1 below.

**Table 1:** Further demographic data of C9orf72 postive Hungarian ALS patients

Male:Female (ratio)	23:40 (0,575)
Mean age at onset in years (SD in years)	57,71 (8,36)
Minimum age at onset (years)	26
Maximum age at onset (years)	72

**Fig. 1:**
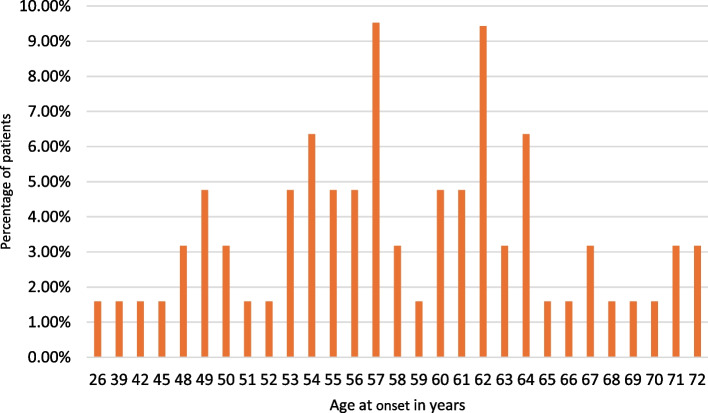
Distribution of age at onset of C9orf72 postive Hungarian ALS patients

The mean age at onset of the cohort was 57,71 years (SD: 8,36 years). Minimum age at onset was 26 years, while the symptoms of the oldest patient started at 72 years old. The median age at onset was 57 years (Fig. 1).

The mean time elapsed from onset of symptoms to the genetic diagnosis was 10,2 months (SD: 9,50 months, median: 8 months). In 22 patients the genetic diagnosis was established in less than 6 months after the onset of symptoms. Currently 12 patients are alive from our cohort. On average patients had a survival of 27,78 months (SD: 14,92 months, 1 st quartile 18 months, 3rd quartile 36 months) from symptom onset. Sex of the patients also influenced survival, male patients had an average survival of 23,69 months (SD: 8,34 months), while female patients had a longer but more scattered mean survival of 2,5 years from the onset of symptoms (mean survival: 29,28 months, SD: 16,78 months). Time to percutaneous endoscopic gastrostomy and time to initiation of invasive ventilation could not be calculated due to the lack of sufficient data from our cohort.

The most common site of onset was bulbar, with 50,79% of patients (32/63) having dysarthria or dysphagia as the presenting symptom. Lower motor neuron (LMN) onset was noted in 28 patients (28/63, 44,44%), while only 3 patients (3/63, 4,76%) had an upper motor neuron (UMN) damage associated presenting symptom.

ALS-FRS-R scores were available in 51 patients, the mean slope of ALS-FRS-R score was −1,37 point/month (SD: 1,32 points/month), with a median value of −1 point/month (1st quartile: −1,67 points/month, 3rd quartile: −0,625 point/month). Slope was retrospectively calculated based on 3,6 recordings on average.

**Fig. 2:**
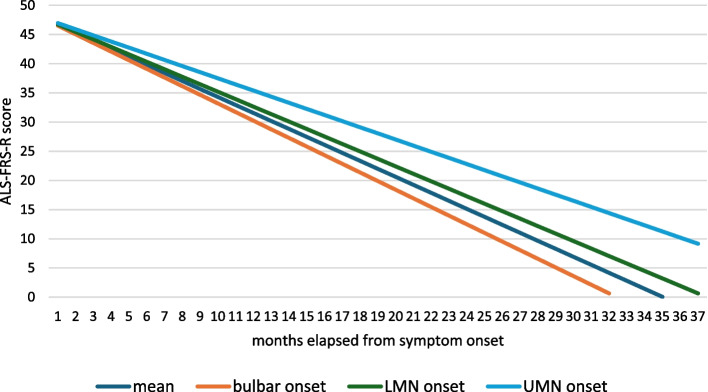
ALS-FRS-R slopes in subgroups based on site of onset of the disease among C9orf72 postive Hungarian ALS patients. The maximum ALS-FRS-R score is 48 and its monthly decline characterizes the progression rate of the disease

When conducting subgroup analyses, the bulbar onset subgroup had the fastest progressing disease, −1,48 points/month decline could be observed in these patients, while LMN and UMN onset patients had a progression rate of −1,28 points/months and −1,05 points/months respectively. When grouping UMN an LMN onset patients as one subgroup, an ALS-FRS-R slope of −1,25 points/month could be calculated (Fig. 2).

**Fig. 3:**
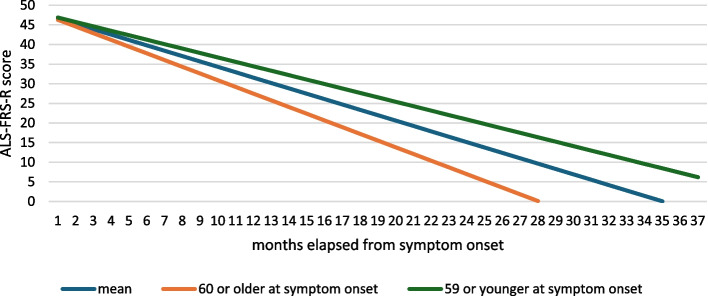
ALS-FRS-R slopes in subgroups based on age at onset of C9orf72 positive ALS patients

ALS-FRS-R score progression was faster in patients with a disease onset at 60 years or later in life, their mean monthly progression was −1,71 points on the ALS-FRS-R scale (*p* = 0.246848) (Fig. 3).

**Fig. 4:**
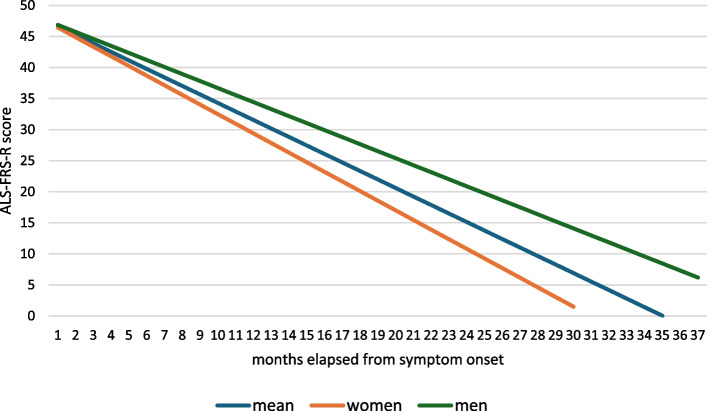
ALS-FRS-R slopes in subgroups based on sex of patients

Males had an average ALS-FRS-R slope of −1,13 points/months (SD: 0,69 point/month), while female *C9orf72* positive ALS patients had an average ALS-FRS-R slope of −1,55 points/month (SD: 1,54 point/month, *p* = 0.069994) (Fig. 4).

The mean ALS-FRS-R slope of patients residing in Eastern Hungarian counties was slightly lower than that of the overall cohort (−1.34 points/month, SD 0.72), whereas patients from Central and Western Hungarian counties had a slightly faster mean progression (−1.38 points/month, SD 1.52). Given the small absolute difference and the limited sample size, this observation may reflect random variation rather than a true regional effect.

**Fig. 5:**
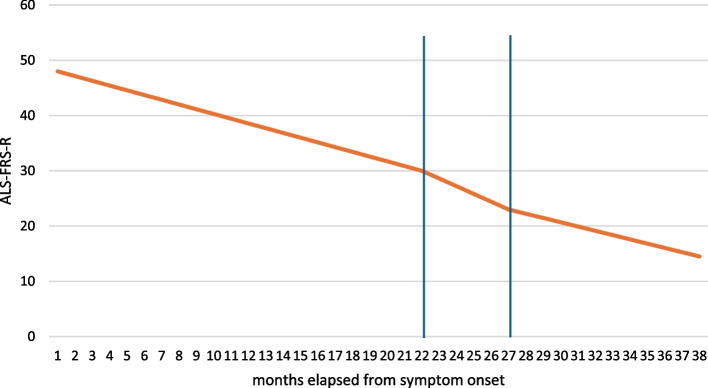
Mean ALS-FRS-R slope of all C9orf72 positive patients in each MITOS stage. Blue vertical lines represent the divide between MITOS satges

MITOS staging could be acquired in case of 51 patients. The mean ALS-FRS-R score at reaching MITOS stage 1 was 30 points (SD: 4,09 points), patients entered stage 1 of MITOS on average 21,04 months (SD: 13,29 months) after the onset of symptoms. MITOS stage 2 was reached on average 26,27 months (SD: 9,57 months) after the first symptoms appeared, patients scored 22,78 points (SD: 4,28 points) on the ALS-FRS-R on average at this timepoint. Patients reached MITOS stage 3 on average 37 months (SD: 12,93 months) after disease onset, their ALS-FRS-R average score was 14,5 points (SD: 3,39 points) at this stage. When calculating mean ALS-FRS-R slope in each MITOS stage, MITOS 1 is associated with −0,86 ALS-FRS-R points/month, while patients in MITOS 2 lose 1,4 points/month on the ALS-FRS-R scale on average. In MITOS 3 the average ALS-FRS-R slope is −0,77 points/month (Fig. 5).

A slightly lower ALS-FRS-R score could be observed in case of patients with a bulbar onset disease when reaching MITOS stage 1 from stage 0, their average ALS-FRS-R score was 28,9 when losing their independence on the first functional domain. Patients with a LMN or UMN onset had a slightly higher ALS-FRS-R score than the whole cohort when entering MITOS stage 1, they scored 30,79 points on average at this time. None of these observations reached the level of significance but showed a clear tendency (*p* = 0.137224). However, none of these tendencies could not be observed at other MITOS stages in any of the subgroups.

Twenty-one patients had reported a positive family history for ALS or FTD (21/63, 33,33%), thus they were categorized as familial ALS cases (fALS). Thirty-four patients had a confirmed negative family history, thus were considered cases of sporadic origin (sALS). No data on family history was available for 8 patients. Mean age at onset did not differ significantly between familial ALS patients and sporadic ALS patients (57,33 years for fALS patients and 57,99 years for sALS patients). Patients with a positive family history had a faster progressive disease compared to patients with a confirmed negative family history or with unkown family history, their mean ALS-FRS-R decline was −1,64 points/month (SD: 1,86 points/month, *p* = 0.150906). Out of the patients who had a positive family history, in 13 cases anticipated disease course was observed (13/21, 61,9%), meaning their onset of disease was earlier than their predecessors’. Patients with an anticipated course of disease had a faster progressing disease compared to the whole cohort and to the positive family history sub-cohort, their mean ALS-FRS-R slope was −1,76 points/months (SD: 2,37 points/month).

Cognitive decline was noted in case of 13 patients (13/62, 20,97%), a formally established diagnosis of frontotemporal dementia was present in 4 cases (4/62, 6,45%) in total. The mean ALS-FRS-R slope of patients with a cognitive decline was −0,72 point/month (SD: 0,35 point/month). A mean Addenbrooke's Cognitive Examination score at diagnosis was 74, while in cases where only Mini Mental State Examination score was available, 24,5 point was the average score at first examination. Table 2 shows the determinants of faster or slower progression of disease in our cohort.

**Table 2:** Determinants of disease progression of C9orf72 positive ALS patients

Mean ALS-FRS-R slope of the cohort	−1,37 point/month (SD: 1,32 points/month)
Determinants of faster disease progression	bulbar onset diease:−1,48 points/month
	female patients:−1,55 points/month
	60 years or older at disease onset:−1,73 points/month
	patients with a positive family history:−1,64 points/month
	patients with anticipated disease:−1,76 points/month
Determinants of slower disease progression	LMN onset disease:−1,28 points/month
	UMN onset disease:−1,05 points/month
	patients with psychiatric comorbidities:−0,93 point/month
	patients with cognitive decline:−0,72 point/month
	male patients:−1,13 points/month

Psychiatric comorbidities were observed in 17 patients (17/63, 26,98%) with affective disorders being the most common (13 patients) and anxiety (5 patients) was the second most common psychiatric symptom in our cohort. Further psychiatric symptoms noted were as follows: a one-time polymorphic psychotic episode at 57 years old (1 patient), gambling addiction (1 patient). One patient may have had multiple psychiatric diagnoses. Patients with psychiatric comorbidities seem to have a slower progressing disease compared to the whole cohort, their mean monthly decline on the ALS-FRS-R scale did not reach −1 point/month (−0,93 point/month, SD: 0,61 point/month) (*p* = 0.418872).

**Fig. 6:**
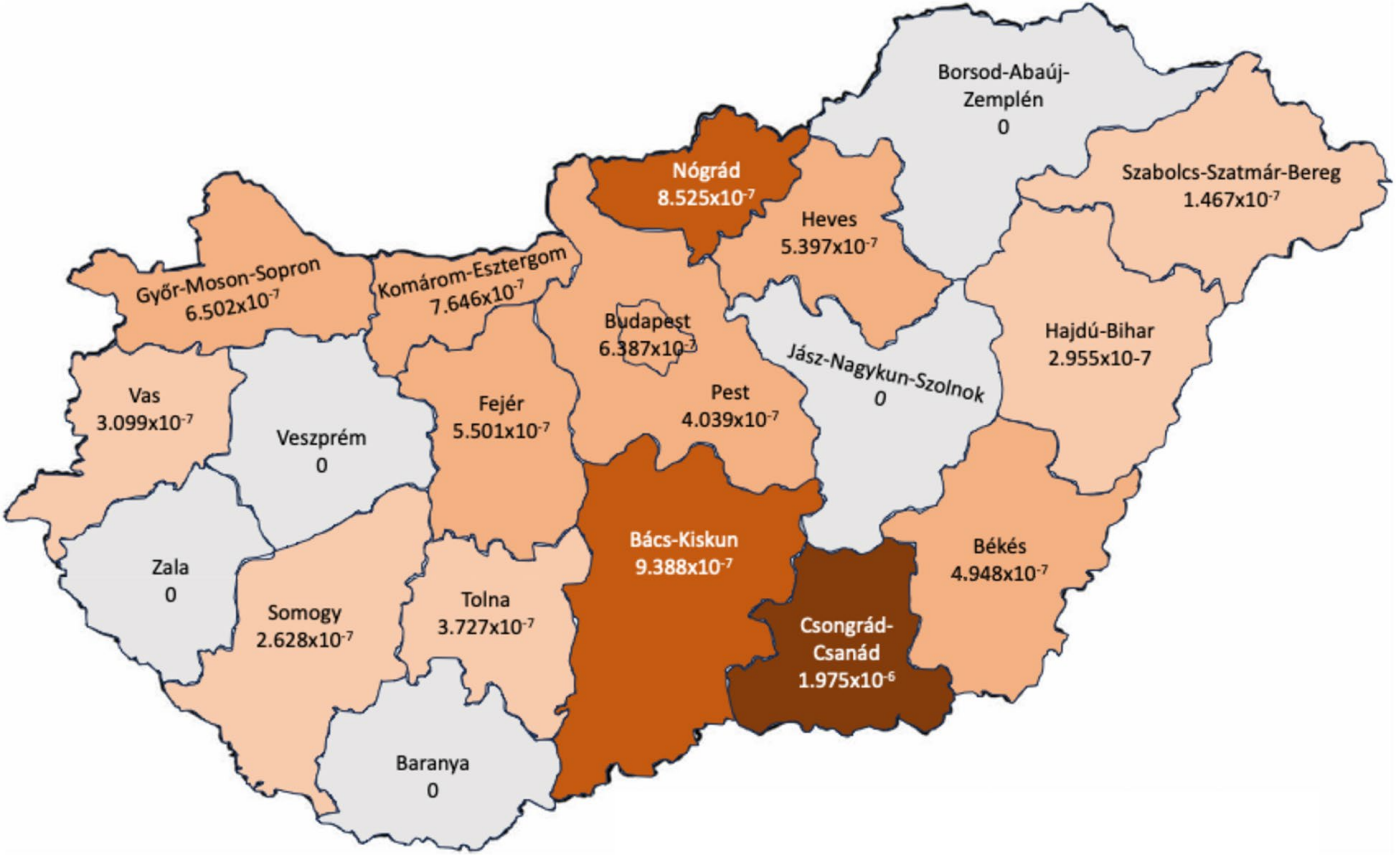
Period prevalence of C9orf72 positive ALS patients 2012–2024 by counties. The darker the colour of the counties, the higher the period prevalence in the are of C9orf72 positive ALS patients. No C9orf72 positive ALS patients were identified in the grey counties during the investigation period

Geographical distribution of *C9orf72* positive ALS patients was determined using their home address at the time of diagnosis. Rate of *C9orf72* positive ALS patients per population was calculated using 2025 data of Hungarian population. At the time of diagnosis 5 patients resided outside of Hungary but were of Hungarian ethnicity and underwent examinations in Hungary in regard to their disease. Four patients stemmed from Serbia and one from Slovakia. Altogether 58 *C9orf72* positive patients residing in Hungary were recorded since 2012, thus the period incidence of *C9orf72* mutation in the last 13 years was 0,000615% (58/9′584′627), meaning 0,61 patient/100 000 people. Fig 6 shows the geographical distribution of *C9orf72* positive ALS patients in Hungary.

The highest period incidence of *C9orf72* positive patients was noted in the Southern part of the country with Csongrád-Csanád and Bács-Kiskun counties having the highest number of *C9orf72* positive patients per population over these 13 years. In five counties (Veszprém, Zala, Baranya, Borsod-Abaúj-Zemplén, Jász-Nagykun-Szolnok) no *C9orf72* positive patients were recorded (Fig. 6).

Patients residing in Eastern Hungary had a mean survival of 21.14 months (SD 7.89), compared with 30.75 months (SD 16.48) in Central and Western Hungary. Although this difference reached nominal statistical significance (p = 0.046, Hedges’ g = 0.67), the small cohort size and large variability suggest that the finding may reflect chance rather than a true regional effect.

All patients and their asymptomatic first-degree relatives were offered genetic counseling to discuss potential risks, testing options, and implications for family planning. Only 5 individuals have requested presymptomatic genetic testing so far. During the family screening we uncovered 5 pre-symptomatic carriers of the *C9orf72* repeat expansion from 3 families.

## Discussion

This study represents the first comprehensive national characterization of *C9orf72* repeat expansion–associated ALS in Hungary. By analyzing 63 genetically confirmed patients and reviewing 959 ALS cases tested since the introduction of *C9orf72* diagnostics in 2011, we estimate that pathogenic *C9orf72* repeat expansions account for 6.57% of ALS cases in the Hungarian population. This figure provides an important baseline for understanding the genetic architecture of ALS in Hungary and places the Hungarian cohort within the lower range of European *C9orf72* carriership frequencies.

A previous Hungarian report identified *C9orf72* repeat expansions in 9.3% of ALS patients (Tripolszki et al. 2019). However, that study examined a smaller and more selected cohort. The lower carriership frequency observed in the present study is most plausibly explained by the substantially larger cohort size and more comprehensive national coverage, rather than by differences in genetic testing indications. Indeed, the inclusion of two diagnostic laboratories and multiple referral centres likely provides a more accurate estimate of the true population frequency.

In a broader European context, the frequency of *C9orf72* repeat expansions among ALS patients varies widely, ranging from approximately 3–4% in Belgian and Hungarian cohorts to over 30% in Finland (Zee et al. 2013; Laaksovirta et al. 2022). Our findings are therefore consistent with a Central–Eastern European position within this spectrum. As reported previously, African populations show carriership frequencies comparable to those in Europe, whereas *C9orf72* repeat expansions are rare in Asian populations, including China, Japan, and Iran (Kacem et al. 2022; Nel et al. 2019; Yang et al. 2022; Alavi et al. 2014; Ogaki et al. 2012). These marked geographic differences likely reflect historical population genetics and founder effects rather than disease-specific mechanisms.

Based on national population data, we show that 58 *C9orf72*-positive ALS patients residing in Hungary were diagnosed between 2012 and 2024, corresponding to an average annual incidence of approximately 0.047 cases per 100,000 inhabitants. This figure is markedly lower than incidence estimates reported from Western European countries (Brown et al. 2021). Rather than reflecting true biological differences, this discrepancy likely indicates underdiagnosis and limited awareness of genetic testing in neurodegenerative diseases within Hungary. In this context, our findings highlight the need for broader and more systematic implementation of genetic testing in ALS.

Notably, more than 90% of patients in our cohort were referred through a rare disease expert centre. This observation underscores the central role of multidisciplinary rare disease centres in the diagnosis, follow-up, and management of ALS, particularly in genetically defined subgroups. As disease-modifying therapies targeting specific genetic forms of ALS become available, such centres will be essential for identifying trial-ready patient populations.

The recent availability of tofersen in Hungary has already led to a substantial increase in *SOD1* genetic testing requests. A similar increase can reasonably be anticipated with the advent of *C9orf72*-targeted therapies, further emphasizing the importance of preparedness within clinical and diagnostic infrastructures.

The clinical phenotype observed in our cohort aligns well with previously published data on *C9orf72*-associated ALS. Bulbar onset was the most frequent initial manifestation, consistent with reports from European and North American cohorts, although lower rates have been described in Chinese and Finnish populations (Chiò et al. 2012; Laaksovirta et al. 2022; Trojsi et al. 2019; Cooper-Knock et al. 2014; Shen et al. 2024). In all patients with concomitant FTD, disease onset was bulbar, supporting previous observations that cognitive involvement increases the likelihood of bulbar presentation (Shen et al. 2024). Older age at onset and bulbar onset were both associated with faster disease progression and shorter survival, in agreement with established literature (Laaksovirta et al. 2022; Trojsi et al. 2019).

Survival analysis revealed a statistically significant difference between patients residing in Eastern Hungary and those from the central and western regions of the country, with a mean survival difference of approximately 9.5 months. Given Hungary’s relatively small geographic size and the limited sample size, this finding should be interpreted with caution. Rather than reflecting a true biological effect, the observed disparity is more likely attributable to regional differences in healthcare access and availability (Dusek et al. 2013). Eastern Hungary is known to be less developed, and disparities in both primary and specialist care have increased in recent years. The paradoxical observation of slower ALS-FRS-R decline but shorter survival in Eastern Hungary further supports the hypothesis that potentially treatable complications may be diagnosed later, leading to premature mortality.

The MITOS staging system allowed longitudinal assessment of functional decline in our cohort (Chiò et al. 2015). While progression from MITOS stage 0 to stage 1 occurred slightly later than reported in a South-Korean ALS cohort, this difference likely reflects cohort composition and genetic homogeneity (He et al. 2021). Importantly, this study is the first to apply MITOS staging in a purely C9orf72-positive ALS cohort. Nevertheless, our findings confirm that MITOS is less sensitive to early functional changes, and ALSFRS-R remains the most informative tool for detecting subtle disease progression (Fang et al. November 2016; Ferraro et al. 2016).

A positive family history of ALS or other neurodegenerative diseases was documented in approximately one-third of patients, a lower proportion than reported in some Italian cohorts (Trojsi et al. 2019). This discrepancy may reflect incomplete family history data, limited awareness of neurodegenerative diagnoses in previous generations, or premature death from unrelated causes.

Cognitive impairment was observed in approximately one-fifth of patients, with clinically diagnosed FTD present in 6.45% of the cohort. These rates are lower than those reported in some Western European and Finnish cohorts and may reflect underdiagnoses rather than true absence of cognitive involvement (Chiò et al. 2012; Laaksovirta et al. 2022; Umoh et al. 2016). Advanced disease stage, anarthria, limited access to neuropsychological testing, and loss to follow-up likely contribute to underrecognition of FTD in routine clinical practice.

Psychiatric comorbidities were present in more than one-quarter of patients, with affective disorders being the most common. While these symptoms may partly reflect psychological responses to a fatal diagnosis, previous studies have demonstrated increased rates of psychiatric disease both before and after ALS diagnosis (Trojsi et al. 2019; Longinetti et al. 2017). Interestingly, in our cohort, cognitive and psychiatric involvement were associated with slower ALSFRS-R decline, possibly reflecting a prolonged prodromal phase during which psychiatric or cognitive symptoms precede overt motor neuron involvement (Hu et al. 2013).

This study has several limitations. Although it represents the largest genetically confirmed *C9orf72*-associated ALS cohort from Hungary to date, the sample size remains modest, and some observed associations—particularly regional survival differences—may reflect random variation. The retrospective design and referral through specialized centers may introduce selection bias, while incidence estimates are influenced by historical variability in genetic testing practices. Cognitive and psychiatric involvement may be under-recognized, and survival analyses did not account for all potential confounders related to healthcare access and comorbidities.

## Conclusion

We present the first comprehensive phenotypic characterization of Hungarian patients with *C9orf72* repeat expansion–associated ALS, based on a nationwide cohort collected over 13 years following the introduction of *C9orf72* testing in Hungary. This dataset provides insight into population-specific features and contributes to a more complete understanding of the clinical and epidemiological heterogeneity of *C9orf72*-associated ALS.

Despite limitations inherent to retrospective data collection and cohort size, this ethnically homogeneous national cohort represents a meaningful contribution given Hungary’s population and complements larger international studies. Systematic characterization and longitudinal follow-up of genetically defined, trial-ready ALS cohorts will be essential as targeted therapies for C9orf72-associated ALS approach clinical implementation.

## Data Availability

The datasets used and/or analysed during the current study are available from the corresponding author on reasonable request.
